# Recombinant Adiponectin Induces the Production of Pro-Inflammatory Chemokines and Cytokines in Circulating Mononuclear Cells and Fibroblast-Like Synoviocytes From Non-Inflamed Subjects

**DOI:** 10.3389/fimmu.2020.569883

**Published:** 2021-02-01

**Authors:** Yuan Zhang, Jonathan Aldridge, Georgios K. Vasileiadis, Helena Edebo, Anna-Karin H. Ekwall, Anna-Carin Lundell, Anna Rudin, Cristina Maglio

**Affiliations:** ^1^Department of Rheumatology and Inflammation Research, Sahlgrenska Academy, University of Gothenburg, Gothenburg, Sweden; ^2^Wallenberg Centre for Molecular and Translational Medicine, University of Gothenburg, Gothenburg, Sweden; ^3^Clinic of Orthopedics, Kungälv Hospital, Kungälv, Sweden

**Keywords:** adiponectin, inflammation, chemokines, cytokines, peripheral blood mononuclear cells, fibroblast-like synoviocytes

## Abstract

Adiponectin is an adipokine with a modulatory role in metabolism and exerting both anti- and pro-inflammatory effects. Levels of adiponectin are increased in serum and synovial fluid from patients with rheumatoid arthritis (RA). Adiponectin is able to stimulate the production of different pro-inflammatory factors from peripheral blood mononuclear cells (PBMCs) and fibroblast-like synoviocytes (FLS) from subjects with established RA. As increased circulating adiponectin levels are a risk factor for future development of RA in subjects with obesity, we hypothesize that adiponectin is implicated in the development of RA at an early stage by initiating the pro-inflammatory processes associated with the disease pathogenesis. Therefore, we aimed to determine if adiponectin is able to induce pro-inflammatory responses in cells involved in the pathogenesis of RA, but collected from subjects without any known inflammatory disease. PBMCs and FLS were obtained from non-inflamed subjects and stimulated with 5 μg/ml human recombinant adiponectin. Supernatants collected after 48 h were analyzed for the production of 13 chemokines and 12 cytokines using multiplex assay and ELISA. Adiponectin significantly stimulated the production of CXCL1, CXCL5, and interleukin (IL)-6 in both PBMCs and FLS, whereas it induced CCL20, CCL4, CCL3, CCL17, tumor necrosis factor (TNF), granulocyte-macrophage colony-stimulating factor and IL-10 only in PBMCs, and CXCL8, CXCL10, CCL5, CCL11, and CCL2 only in FLS. Pre-stimulation with TNF of FLS from non-inflamed subjects did not significantly enhance the release of most pro-inflammatory factors compared to adiponectin alone. Our findings indicate that PBMCs and FLS from non-inflamed subjects react to adiponectin stimulation with the secretion of several pro-inflammatory chemokines and cytokines. These results suggest that adiponectin is able to initiate pro-inflammatory responses in cells from non-inflamed subjects and support the hypothesis that adiponectin is implicated in the early phases of RA pathogenesis.

## Introduction

Adiponectin is a cytokine mainly secreted by the adipose tissue, a so-called adipokine (1, 2). Although mostly produced by the white adipose tissue, serum adiponectin levels are surprisingly low in subjects with obesity and high adiponectin levels associate with a beneficial metabolic profile (2, 3). Adiponectin is involved in glucose and lipid metabolism and plays a role in inflammation (4, 5). Since identified in the 1990s (1), adiponectin have been considered mostly as an anti-inflammatory adipokine, being able to induce the production of interleukin (IL)-10 and reduce tumor necrosis factor (TNF) in macrophages (6, 7). However, adiponectin belongs to a family of proteins with pro-inflammatory functions, and shares structure homologies with both TNF and immune complement protein C1q (1, 8). In fact, adiponectin also has pro-inflammatory effects as it is able to promote pro-inflammatory responses in CD4^+^ T cells and macrophages, such as IFN-γ and TNF secretion, respectively (9). Furthermore, adiponectin induces the production of IL-6, TNF, CXCL1, and CXCL8 in total T- and B-lymphocytes from healthy subjects (10).

Adiponectin levels are elevated in both serum and synovial fluid from subjects with rheumatoid arthritis (RA), a chronic systemic inflammatory disease (11–15). We have also recently shown that elevated serum adiponectin levels precede the development of RA in a cohort of subjects with obesity (16). However, it is unclear if the increase in adiponectin levels before the onset of the first symptoms is pathogenic in the context of RA or if it is a counterbalance mechanism. Although RA can affect different non-articular organs, the disease is typically characterized by synovial inflammation of diarthrodial joints, leading to progressive joint damage (17). Pro-inflammatory chemokines and cytokines secreted by fibroblast-like synoviocytes (FLS), lymphocytes, and macrophages play an important role in the pathogenesis of RA (18, 19). Adiponectin stimulation of FLS from patients with RA is able to induce the production of IL-6, CXCL8 and prostaglandin E_2_, which supports the hypothesis that adiponectin exerts pro-inflammatory functions in RA (20, 21).

The development of RA includes a preclinical period when arthritis is not clinically detectable, but the interaction between genetic and environmental factors and the production of RA-related autoantibodies and inflammatory biomarkers have already occurred (22, 23). Plasma levels of adiponectin have been shown to associate with levels of circulating pro-inflammatory cytokines, i.e., IL-2, IL-6, IL-9, and granulocyte-macrophage colony-stimulating factor (GM-CSF) in relatives of RA patients that had a high-risk autoantibody profile (24). Together with our finding that an increase in serum adiponectin levels predicts the development of RA, this suggests that adiponectin plays a role in the initiation of the disease (16). In the current study, we aimed to determine if adiponectin is able to induce pro-inflammatory responses in cells involved in the pathogenesis of RA, but collected from subjects without any known inflammatory disease. Our results show that adiponectin stimulates the production of several pro-inflammatory chemokines and cytokines in both PBMCs and FLS from non-inflamed subjects. These findings support the hypothesis that adiponectin has pro-inflammatory functions that may be implicated in the initiation of RA.

## Methods

### Cell Isolation and Cell Culture

Human PBMCs were isolated from whole blood sampled from eight non-inflamed subjects (three males and five females, median age 43, ranging from 25 to 72 years) and four subjects with RA (two males and two females, median age 65, ranging from 37 to 72 years, two seronegative, two rheumatoid factor and/or anti-citrullinated protein antibody positive) using Lymphoprep™ (Axis-Shield, Oslo, Norway) density gradient centrifugation. Isolated PBMCs were frozen in heat inactivated Gibco™ Fetal Bovine Serum supplemented with 7.5% Dimethyl sulfoxide at -80 °C until usage.

FLS from non-inflamed subjects were isolated from synovial tissues obtained from nine patients without previous history of arthritis who underwent diagnostic arthroscopy due to a previous injury that happened more than 2 months before (five males and four females, median age 29, ranging from 18 to 70 years). At the time of the examination, the joints did not display any sign of macroscopic inflammation or swelling. FLS from subjects with RA were isolated from synovial tissue specimens, obtained during joint replacement surgery at Sahlgrenska University Hospital in Sweden (two males and one female, median age 50, ranging from 27 to 63 years, all rheumatoid factor and/or anti-citrullinated protein antibody positive). All RA patients fulfilled the American College of Rheumatology 1987 revised criteria for the disease (25). Representative tissue pieces were minced, treated with 1 mg/ml collagenase/dispase (Roche, Mannheim, Germany) for 1 h at 37°C and then passed through a cell strainer. The cell suspension was rinsed twice in phosphate buffered saline, re-suspended in FLS growth medium (Dulbecco’s modified Eagle’s medium GlutaMAX™ supplemented with 10% heat inactivated Gibco™ Fetal Bovine Serum, 50 μg/ml Gibco™ Gentamicin and 100 U/ml Gibco™ Penicillin-Streptomycin) and incubated at 37°C with 5% CO_2_. All cell culture reagents were purchased from Thermo Fisher Scientific (Waltham, MA, USA) unless otherwise specified. All study participants gave their written informed consent, and the protocol was approved by the regional ethics committee (Dnr 1087-16, Dnr 573-07, and Dnr 459-18).

### Adiponectin Stimulation

PBMCs were thawed and then rinsed in X-VIVO™ 15 Serum-free Hematopoietic Cell Medium (Lonza, Basel, Switzerland), and 1×10^5^ cells/well were seeded into U-bottom shaped 96-well plates in 100 μl X-VIVO™ 15 medium. PBMCs were stimulated using 0.5, 1.0 or 5.0 μg/ml recombinant human adiponectin protein (Cat. Ab51924, Abcam, Cambridge, UK). The mitogen phytohemagglutinin (PHA; Thermo Fisher Scientific) at 2.5 μg/ml was used as a positive control to activate the PBMCs. The cells were incubated at 37°C with 5% CO_2_, and the supernatants were collected after 6, 12, 24, or 48 h of stimulation. Based on the curves of time- and dose-dependent experiments (**Supplementary Figure 1**), stimulation with 5 μg/ml adiponectin for 48 h were chosen. We confirmed that the recombinant adiponectin was not contaminated by lipopolysaccharides (LPS) with a pilot experiment comparing the pattern of IL-6 production induced by adiponectin and LPS (data not shown).

FLS (passage 4–7) were detached using 0.05% Gibco™ Trypsin-EDTA (Thermo Fisher Scientific), and 5,000 cells/well were seeded into each well of flat bottom shaped 96-well plates in 100 μl FLS growth medium. The cells were incubated at 37°C with 5% CO_2_ for 20 h, and then the FLS growth medium was replaced by 100 μl X-VIVO™ 15 medium and the cells were serum-starved at 37°C with 5% CO_2_ for 4 h. FLS were stimulated using 5.0 μg/ml recombinant adiponectin and 10 ng/ml TNF (Sigma, St. Louis, MO, USA) was used as a positive control to activate the FLS. The cells were incubated at 37°C with 5% CO_2_, and supernatants were collected after 48 h of stimulation. For pre-stimulation, FLS were first stimulated using 10 ng/ml TNF for 24 h, and then 5.0 μg/ml recombinant adiponectin was added after media replacement, the cells were further stimulated for 48 h.

### LEGENDplex™ Bead-Based Immunoassay

In supernatants from PBMCs and FLS, concentrations of the following human chemokines were measured using LEGENDplex™ Human Proinflammatory Chemokine Panel (BioLegend, San Diego, USA): CCL2 (monocyte chemoattractant protein 1; MCP-1), CCL5 (regulated on activation, normal T cell expressed and secreted, RANTES), CXCL10 (10 kDa interferon-gamma-induced protein; IP-10), CCL11 (Eotaxin), CCL17 (thymus- and activation-regulated chemokine; TARC), CCL3 (macrophage inflammatory protein-1α; MIP-1α), CCL4 (MIP-1β), CXCL9 (monokine induced by gamma interferon; MIG), CCL20 (MIP-3α), CXCL5 (epithelial-derived neutrophil-activating peptide 78; ENA-78), CXCL1 (growth-regulated oncogene alpha; GROα), CXCL11 (interferon-inducible T-cell alpha chemoattractant; I-TAC) and CXCL8 (IL-8). Concentrations of the following human cytokines were measured using LEGENDplex™ Human Inflammation Panel (BioLegend): IL-1β, interferon (IFN)-α2, IFN-γ, TNF, IL-6, IL-10, IL-12p70, IL-17A, IL-18, IL-23, and IL-33. All the experiments were performed following manufacturer’s instructions. The samples were read using BD FACSverse Flow Cytometer (BD Biosciences, San Jose, CA, USA), and the data were analyzed using LEGENDplex™ Data Analysis Software V8.0 (BioLegend). Two samples of CXCL1 and one sample in CXCL5 from the adiponectin groups were above the detection ranges, and the values were set to the detection limit.

### Enzyme-Linked Immunosorbent Assay (ELISA)

CXCL8, TNF, IL-1β, IL-6, IL-10, and GM-CSF in supernatants from PBMCs and concentration of IL-6 and GM-CSF in supernatants from FLS was measured using Duoset ELISA kits purchased from Bio-Techne (Minneapolis, MN, USA) following manufacturer’s instructions. The plates were read using micro plate reader CLARIOstar (BMG LABTECH, Offenburg, Germany) at 450 nm with wavelength correction at 540 nm.

### Statistical Analysis

All the statistical analyses were performed using Prism 8 software (GraphPad Software Inc., La Jolla, CA, USA) and SPSS Statistics 25 (IBM, Armonk, NY, USA). All the data are presented as individual values and medians, and statistical significance was determined as *P* value ≤ 0.05 by Friedman’s test with post-hoc analysis unadjusted for multiple tests (labelled *), Kruskal-Wallis test with post-hoc analysis unadjusted for multiple tests (labelled #) or two-way analysis of variance (ANOVA) with Dunnett’s test.

## Results

### Profile of Chemokines Produced by Adiponectin-Stimulated PBMCs From Non-Inflamed Subjects

**Figure 1:**
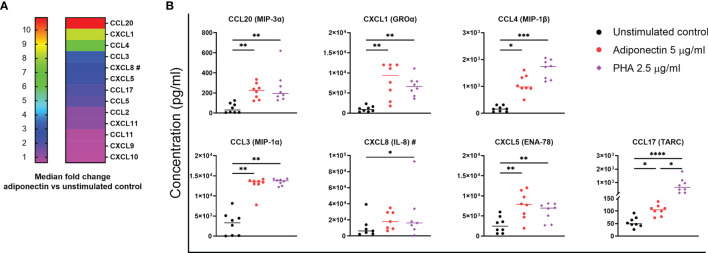
Profile of chemokines produced by adiponectin-stimulated peripheral blood mononuclear cells (PBMCs) from non-inflamed subjects. Chemokine levels in supernatants from PBMCs at 48 h after adiponectin stimulation were measured using Legendplex assay or ELISA (#). **(A)** Median fold changes of chemokine levels after adiponectin stimulation compared to unstimulated controls. **(B)** Levels of CCL20, CXCL1, CCL4, CCL3, CXCL8, CXCL5, and CCL17 in supernatants from unstimulated controls, adiponectin- or PHA-stimulated cells. The results are shown as the individual values and medians from 7–8 healthy donors. Significance was determined using Friedman’s test with post-hoc analysis unadjusted for multiple tests, *P ≤ 0.05, **P ≤ 0.01, ***P ≤ 0.001, ****P ≤ 0.0001.

We first screened the profile of chemokines produced by PBMCs from non-inflamed subjects stimulated with 5.0 µg/ml adiponectin for 48 h. **Figure 1A** depicts the median fold changes of chemokine levels in supernatant after adiponectin stimulation compared to unstimulated controls. CCL20 (11-fold increase), CXCL1 (8-fold), CCL3 (3-fold), and CXCL5 (2-fold) were significantly upregulated by adiponectin, and the effect of adiponectin was similar to or greater than the effect of PHA (**Figure 1B**). Adiponectin significantly induced the production of CCL4 (6-fold) and CCL17 (2-fold) as well, although the effect was lower than PHA stimulation (**Figure 1B**). The induced production of these chemokines was time- and dose-dependent (**Supplementary Figures 1A–D, F** and **G**). The induction of CXCL8 (2-fold) was not significant at 48 h and did not follow a time- or dose-dependent manner, but the levels of CXCL8 were significantly elevated by 5 µg/ml adiponectin at 6 and 12 h (*P* = 0.0015 and 0.0154 respectively; **Supplementary Figure 1E**). These results illustrate that adiponectin is able to induce the production of pro-inflammatory chemokines in PBMCs from non-inflamed subjects.

### Profile of Cytokines Produced by Adiponectin-Stimulated PBMCs From Non-Inflamed Subjects

**Figure 2:**
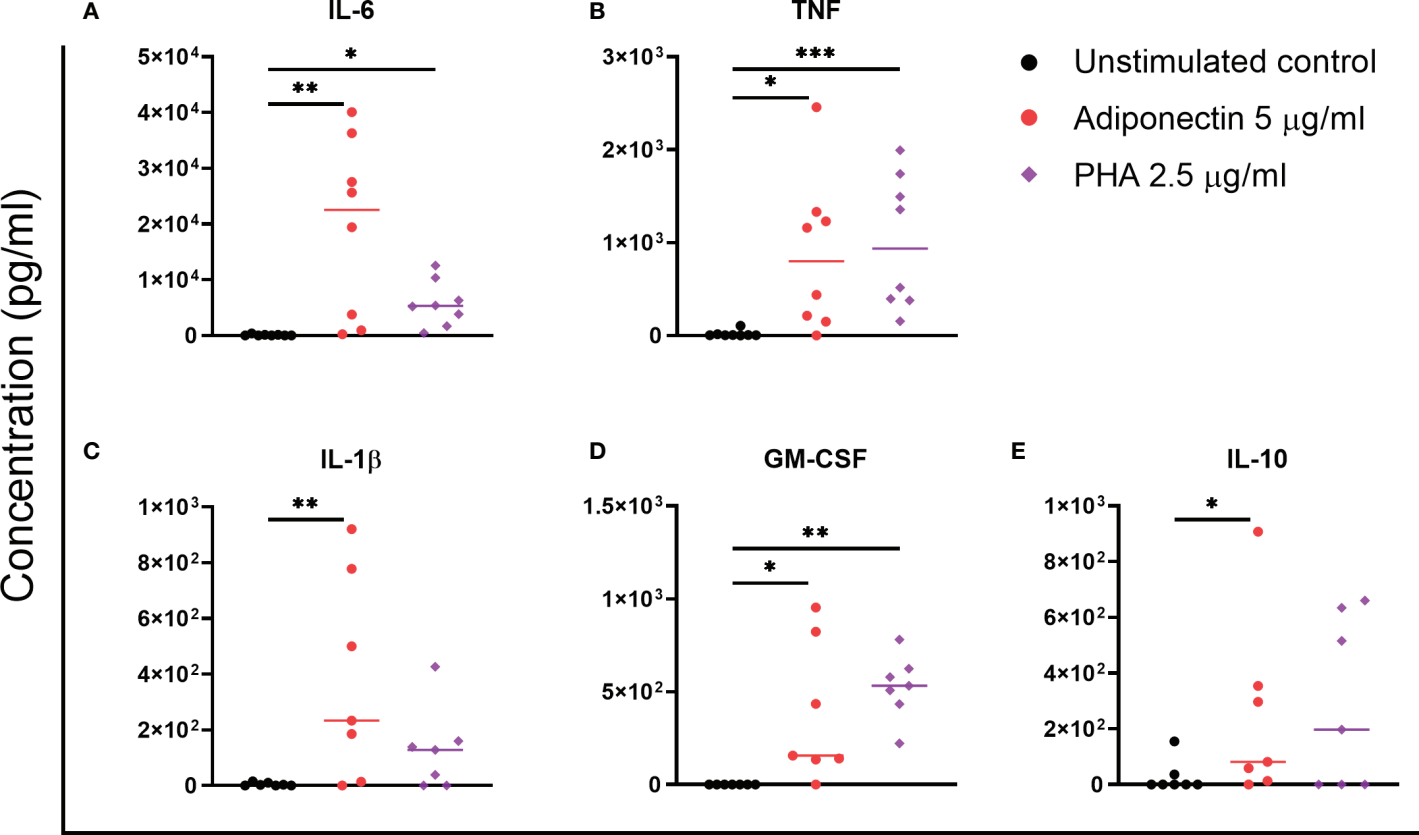
Profile of cytokines produced by adiponectin-stimulated peripheral blood mononuclear cells (PBMCs) from non-inflamed subjects. Levels of IL-6 **(A)**, TNF **(B)**, IL-1β **(C)**, GM-CSF **(D)**, and IL-10 **(E)** in supernatants from healthy PBMCs at 48 h after adiponectin or PHA stimulation were measured using ELISA. The results are shown as the individual values and medians from 7–8 healthy donors. Significances were determined using Friedman’s test with post-hoc analysis unadjusted for multiple tests, *P ≤ 0.05, **P ≤ 0.01, ***P ≤ 0.001.

We further screened the profile of cytokines produced by adiponectin-stimulated PBMCs from non-inflamed subjects. Only a few cytokines were detectable in supernatant using the LEGENDplex™ bead-based immunoassay, i.e., IL-1β, IL-6, IL-10, and TNF. The concentrations of these cytokines were confirmed using ELISA and are presented in **Figure 2**. GM-CSF, which was not included in the LEGENDplex™ kit, was measured using ELISA. In supernatant from PBMCs, the concentrations of IL-6, TNF, IL-1β, GM-CSF, and IL-10 were significantly increased by adiponectin stimulation compared to the unstimulated controls (**Figure 2**). The changes of IL-6, TNF, and GM-CSF were time- and dose-dependent, the changes of IL-1β was dose-dependent, and the changes of IL-10 was time-dependent (**Supplementary Figure 2A–E**). These results indicate that adiponectin is able to induce several pro-inflammatory cytokines in PBMCs from non-inflamed subjects.

### Profile of Chemokines Produced by Adiponectin-Stimulated FLS From Non-Inflamed Subjects

**Figure 3:**
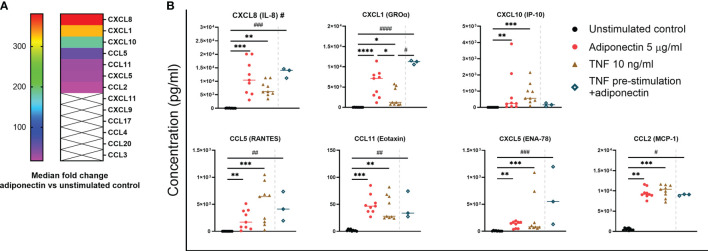
Profile of chemokines produced by adiponectin-stimulated fibroblast-like synoviocytes (FLS) from non-inflamed subjects after stimulation. Chemokine levels in supernatants from FLS at 48 h after adiponectin or tumor necrosis factor (TNF) stimulation were measured using Legendplex assay. FLS in the pre-stimulation group were stimulated by TNF for 24 h, and then the media were replaced, adiponectin was added for a further 48-h stimulation. **(A)** Median fold changes of chemokine levels after adiponectin stimulation compared to unstimulated controls. Chemokines marked with × were either undetectable or the levels were low (< 30 pg/ml). **(B)** Levels of CXCL8, CXCL1, CXCL10, CCL5, CCL11, CXCL5, and CCL2 in supernatants from unstimulated, adiponectin-stimulated, TNF-stimulated and pre-stimulated groups. The results are shown as the individual values and medians from 3 or 9 patients without inflammatory joint disease who underwent diagnostic arthroscopy. Significance among unstimulated, adiponectin-stimulated and TNF-stimulated groups was determined using Friedman’s test with post-hoc analysis unadjusted for multiple tests, *P ≤ 0.05, **P ≤ 0.01, ***P ≤ 0.001, ****P ≤ 0.0001, and the significance between pre-stimulated group and other groups was determined using Kruskal-Wallis test with post-hoc analysis unadjusted for multiple tests, ^#^*P* ≤ 0.05, ^##^*P* ≤ 0.01, ^###^*P* ≤ 0.001, ^####^*P* ≤ 0.0001.

We next investigated the profile of chemokines produced by adiponectin-stimulated FLS collected from nine patients without inflammatory joint disease who underwent diagnostic arthroscopy. **Figure 3A** shows the median fold changes of chemokine levels after stimulation with 5.0 µg/ml adiponectin for 48 h compared to unstimulated controls. The production of CXCL8 (381-fold increase), CXCL1 (328-fold), CXCL10 (185-fold), CCL5 (54-fold), CCL11 (29-fold), CXCL5 (26-fold), and CCL2 (17-fold) were significantly induced by adiponectin in FLS and the effects were either greater or similar to the positive control TNF (**Figure 3B**). CXCL11, CXCL9, CCL17, CCL4, CCL20, and CCL3 were either undetectable or the levels were low (< 30 pg/ml, marked with crosses in the heat map, **Figure 3A**). Our results show that adiponectin is able to induce considerable levels of pro-inflammatory chemokines in FLS from non-inflamed subjects.

TNF is one of the key cytokines responsible for synovial pro-inflammatory responses associated with RA and is also an important target for anti-rheumatic drugs TNF (26). We performed pre-stimulation experiment using FLS from non-inflamed subjects pre-stimulated by TNF to mimic an acute inflammatory condition, and the cells were further stimulated by adiponectin after medium replacement. In this small-scale experiment (n=3), pre-stimulation with TNF before stimulation with adiponectin did not seem to significantly enhance the release of chemokines compared to adiponectin alone but the increase of CXCL1 was significant compared to TNF stimulation alone (**Figure 3B**). These results suggest that adiponectin exhibits a pro-inflammatory profile under a TNF-induced acute inflammatory condition.

### IL-6 Production in Adiponectin-Stimulated FLS From Non-Inflamed Subjects

**Figure 4:**
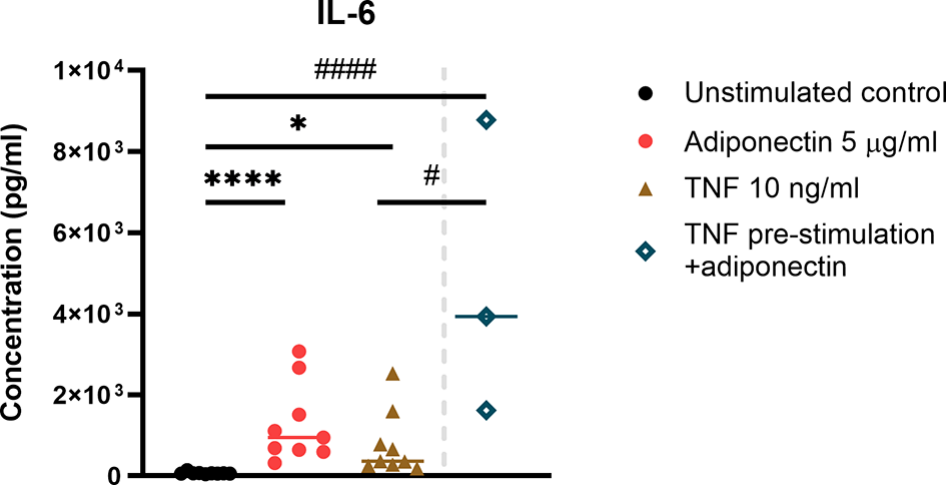
IL-6 production in adiponectin-stimualted fibroblast-like synoviocytes (FLS) from non-inflamed subjects. IL-6 was the only detectable cytokine in supernatants from FLS, and its levels were measured using ELISA at 48 h after adiponectin or tumor necrosis factor (TNF) stimulation. FLS in the pre-stimulation group were stimulated by TNF for 24 h, and then the media were replaced, adiponectin was added for a further 48-h stimulation. The results are shown as the individual values and medians from 3 or 9 patients without inflammatory joint disease who underwent diagnostic arthroscopy. Significance among unstimulated, adiponectin-stimulated and TNF-stimulated groups was determined using Friedman’s test with post-hoc analysis unadjusted for multiple tests, *P ≤ 0.05, ****P ≤ 0.0001, and the significance between pre-stimulated group and other groups was determined using Kruskal-Wallis test with post-hoc analysis unadjusted for multiple tests, #*P* ≤ 0.05, ####*P* ≤ 0.0001.

In FLS from non-inflamed subjects, IL-6 was the only cytokine that can be detected from the supernatant. Adiponectin was able to induce the production of IL-6 in FLS, and the inducing effect was comparable to the positive control TNF (**Figure 4**). In this small-scale experiment (n=3), pre-stimulation with TNF followed by adiponectin enhanced the production of IL-6 compared to stimulation with TNF alone but not compared to adiponectin alone (**Figure 4**). These findings indicate that adiponectin is able to induce the production of the pro-inflammatory cytokine IL-6 in FLS.

### Profile of Chemokines and Cytokines Produced by Adiponectin-Stimulated Human PBMCs and FLS From Subjects With Established RA

Previous studies have shown that adiponectin stimulates pro-inflammatory responses in PBMCs and FLS from subjects with established RA (20, 21). Our current results indicate that adiponectin is also able to stimulate the production of a large variety of chemokines and cytokines from PBMCs and FLS from non-inflamed subjects. As a comparison, we analyzed the profile of chemokine and cytokine production from PBMCs and FLS from a small group of subjects with established RA. In PBMCs from RA subjects, adiponectin induced similar patterns of chemokine and cytokine production compared to PBMCs from non-inflamed subjects as shown in **Supplementary Figure 3A **and** B**, although not all the differences were statistically significant. In FLS from subjects with RA, as in those from non-inflamed subjects, adiponectin significantly induced the production of CXCL8, CXCL1, CXCL10, and IL-6, and the patterns of CCL5, CXCL5, CCL2, and CCL11 stimulation were also similar to those in FLS from non-inflamed subjects (**Supplementary Figure 4**).

## Discussion

Adiponectin is an adipokine with regulatory properties in both metabolism and inflammation (4, 5). The increase in adiponectin levels under inflammatory conditions, such as serum and synovial fluid from subjects with RA, has raised the hypothesis that this adipokine exerts pro-inflammatory functions together with the already known anti-inflammatory effects described in literature (6, 7, 11, 13). As a support for this hypothesis, adiponectin has been shown to induce the production of IL-6, TNF, CXCL1, and CXCL8 in human lymphocytes from healthy subjects (10), and IL-6 and CXCL8 in FLS collected from subjects with RA (20). We have also recently shown that increased circulating adiponectin levels precede the development of RA in subjects with obesity, suggesting that adiponectin could play a role in the early phases of disease pathogenesis (16). The current study shows that adiponectin stimulates the production of a wide range of pro-inflammatory chemokines and cytokines in PBMCs and FLS collected from non-inflamed subjects, thus suggesting that adiponectin is able to initiate pro-inflammatory responses in healthy cells.

The aim of this study was to investigate the effect of adiponectin on cells involved in the pathogenesis of RA, but collected from subjects without any known inflammatory disease. Another study previously screened the profile of chemokines and cytokines produced by adiponectin-stimulated RA effector cells showing that the production of CXCL8, CCL5, TNF, and IL-6 by lymphocytes from healthy subjects, and CXCL1, CXCL5, CXCL8, CCL2, and IL-6 by FLS from patients with RA were significantly increased after adiponectin stimulation (10). Compared to this study, we observed that a similar, but a wider range of chemokines and cytokines could be induced after adiponectin stimulation. However, the cells used in the above-mentioned study were lymphocytes from healthy subjects and FLS from subjects with RA making it difficult to compare their results with our own. To the best of our knowledge, we are the first to investigate how FLS from non-inflamed subjects response to the stimulation with adiponectin. Other studies on related topics focused on FLS from patients with RA and used FLS from subjects with osteoarthritis as controls (20, 27, 28). However, osteoarthritis is an inflammatory joint disease and therefore cannot represent non-inflamed conditions (29, 30). By using PBMCs and FLS from non-inflamed subjects, we set a baseline for understanding the functions of adiponectin at a pre-RA stage.

Our results show that adiponectin induced a different profile of chemokines in PBMCs compared to FLS. We measured 13 inflammatory chemokines, as well as 14 cytokines (Th1, Th2, and Th17) using two multiplex kits and ELISA. The multiplex kits covered a wide range of chemokines and cytokines that are involved in the development of inflammatory diseases such as RA, and that have been previously reported as induced by adiponectin in FLS from patients with RA (10, 20, 28). In both PBMCs and FLS, adiponectin stimulated the production of CXCL1 and CXCL5, whose levels are known to be elevated in serum and synovial fluids of patients with RA compared to controls (31–33). Moreover, adiponectin was able to induce the production of CCL20, CCL4, CCL3, and CCL17 in PBMCs but not in FLS. CCL3, CCL4, and CCL20 exert chemotactic properties by attracting monocytes to the inflamed joints (34, 35). On the other hand, adiponectin stimulated the production of CXCL8, CXCL10, CCL5, CCL11, and CCL2 only in FLS. CXCL8 and CCL2 are pro-inflammatory chemokines elevated in synovial fluids of patients with RA (36, 37). CCL5 and CXCL10 regulate homing and migration of T cells (38, 39). CXCL10 is also able to recruit other immune cells including monocytes, natural killer cells and dendritic cells, and it is a disease activity marker of untreated early RA (39). CCL11 is up-regulated in pre-RA patients as compared to matched healthy controls (40, 41). Interestingly, the fold changes of adiponectin-induced chemokines in FLS were much larger than in PBMCs.

In this study, we observed that adiponectin was able to induce the production of IL-6 in both PBMCs and FLS from non-inflamed subjects, and the production of TNF, IL-1β, GM-CSF, and IL-10 only in PBMCs. IL-6, TNF, IL-1β, and GM-CSF are involved in RA pathogenesis by promoting inflammation and bone degradation. They are also able to induce the production of pro-inflammatory chemokines in the joints (26, 42–45). Adiponectin also stimulated the production of the anti-inflammatory cytokine IL-10 in PBMCs, but the levels of IL-10 were lower than other cytokines, and the induction was only time- but not dose-dependent. The induction of IL-10 by adiponectin might be a regulatory action to counterbalance the systemic inflammation as shown in other contexts (46, 47).

To mimic an acute inflammatory state, we performed pre-stimulation experiments using FLS from non-inflamed subjects. In TNF-pre-stimulated FLS, adiponectin was able to further enhance the production of CXCL1 and IL-6 compared to cells that were only stimulated by TNF, suggesting that adiponectin has pro-inflammatory effects in an acute inflammatory milieu. Adiponectin induced similar responses in cells from subjects with RA and cells from non-inflamed subjects, although the changes of most chemokines and cytokines did not achieve significance in RA cells due to the small sample size. These results support our hypothesis that adiponectin is a pro-inflammatory factor in the context of RA, and that it is able not only to maintain a chronic inflammatory state, but also to initiate it in previously non-inflamed cells. In fact, we could show that the pro-inflammatory effects of adiponectin are not specific for cells from patients with RA. A pre-activation of the cells through a pro-inflammatory environment simulated by TNF is not required for the pro-inflammatory effects of adiponectin itself. Taken all together, our results suggest that adiponectin exerts pro-inflammatory functions both systemically and locally.

In this study, we performed *in vitro* experiments using X-VIVO™ 15 Serum-free Hematopoietic Cell Medium. We decided against using serum-containing medium as adiponectin is known to bind to albumin and may interact with different growth factors (48, 49). Serum may also contain soluble CD14 and LPS-binding protein, which intensify LPS-induced FLS activation in cell culture at low concentrations (50). A pilot experiment performed in FLS showed that those cells grew well in X-VIVO™ 15 medium and responded to stimulation with adiponectin in a similar way as the Dulbecco’s modified Eagle’s medium containing 1.5% heat inactivated fetal bovine serum.

The downstream signaling of adiponectin is mainly mediated by two receptors, AdipoR1 and AdipoR2, which lead to the activation of AMP-activated kinase (AMPK) and peroxisome proliferator-activated receptor signaling pathways, respectively. Both signaling pathways regulate glucose and lipid metabolism and lead to anti-inflammatory responses (51, 52). It has previously been suggested that the adiponectin-induced IL-6 expression in FLS from patients with RA is mediated by AdipoR1 *via* AMPK, p38 and nuclear factor kappa-light-chain-enhancer of activated B cells (28). It is still unknown if all the pro-inflammatory effects of adiponectin go through the same signaling pathway and if the regulation is cell type-specific. The differences in the expression of adiponectin receptors and the activation of downstream factors may explain why the profiles of chemokines and cytokines induced by adiponectin are different in PBMCs and FLS.

Adiponectin circulates in blood in three oligomeric forms: low molecular weight trimers, middle molecular weight hexamers, and high molecular weight multimers, consisting of 12 or more monomers, which may exert different biological functions (53). In this study, we used total adiponectin, including all the forms of the protein. However, the proportion of each adiponectin oligomer in the commercial recombinant protein could differ from the one present in blood thus affecting the results. Further studies using stimulation with different adiponectin forms are needed to give a full characterisation of the profile of chemokines and cytokines produced by adiponectin-stimulated human PBMCs and FLS.

In conclusion, we reported that adiponectin has pro-inflammatory properties by inducing the production of several chemokines and cytokines in PBMCs and FLS from non-inflamed subjects. Our findings provide new understanding of the role of adiponectin in modulating inflammatory networks involved in the pathogenesis of RA. Moreover, the fact that adiponectin is able to induce such changes in cells from non-inflamed subjects supports the hypothesis that adiponectin is implicated in the early phases of RA pathogenesis. Further *in vitro* studies in cells from both non-inflamed subjects and patients with RA are needed to better characterize the mechanisms by which adiponectin might contribute to the pathogenesis of RA.

## Data Availability Statement

The original contributions presented in the study are included in the article/**Supplementary Material**. Further inquiries can be directed to the corresponding author.

## Ethics Statement

The studies involving human participants were reviewed and approved by Regional Ethics Review Board in Gothenburg. The patients/participants provided their written informed consent to participate in this study.

## Author Contributions

YZ and CM designed the study and wrote the manuscript. YZ, JA, and GV performed the experiments. JA, HE, A-KE, A-CL, and AR were involved in collecting samples. YZ, A-KE, A-CL, AR, and CM contributed to the interpretation of the results. CM supervised all aspects of the work. All authors contributed to the article and approved the submitted version.

## Funding

This work was supported by the Knut and Alice Wallenberg Foundation and the Wallenberg Centre for Molecular and Translational Medicine at the University of Gothenburg (grant 2016/4839), Sweden to CM. This study was also supported by grants from the Swedish Research Council (grant 2016–01574) and the Region Västra Götaland (agreement concerning research and education of doctors; ALF; grant ALFGBG-143331) to AR.

## Conflict of Interest

The authors declare that the research was conducted in the absence of any commercial or financial relationships that could be construed as a potential conflict of interest.
