# Supplementary material for: Recombinant Adiponectin Induces the Production of Pro-Inflammatory Chemokines and Cytokines in Circulating Mononuclear Cells and Fibroblast-Like Synoviocytes From Non-Inflamed Subjects
Source: Front Immunol. 2021 Feb 1;11:569883. doi: 10.3389/fimmu.2020.569883 (PMC7882698; doi:10.3389/fimmu.2020.569883)
Supplement: Supplementary file 1 [file DataSheet_1.docx]

Supplementary Material

# Supplementary Figures
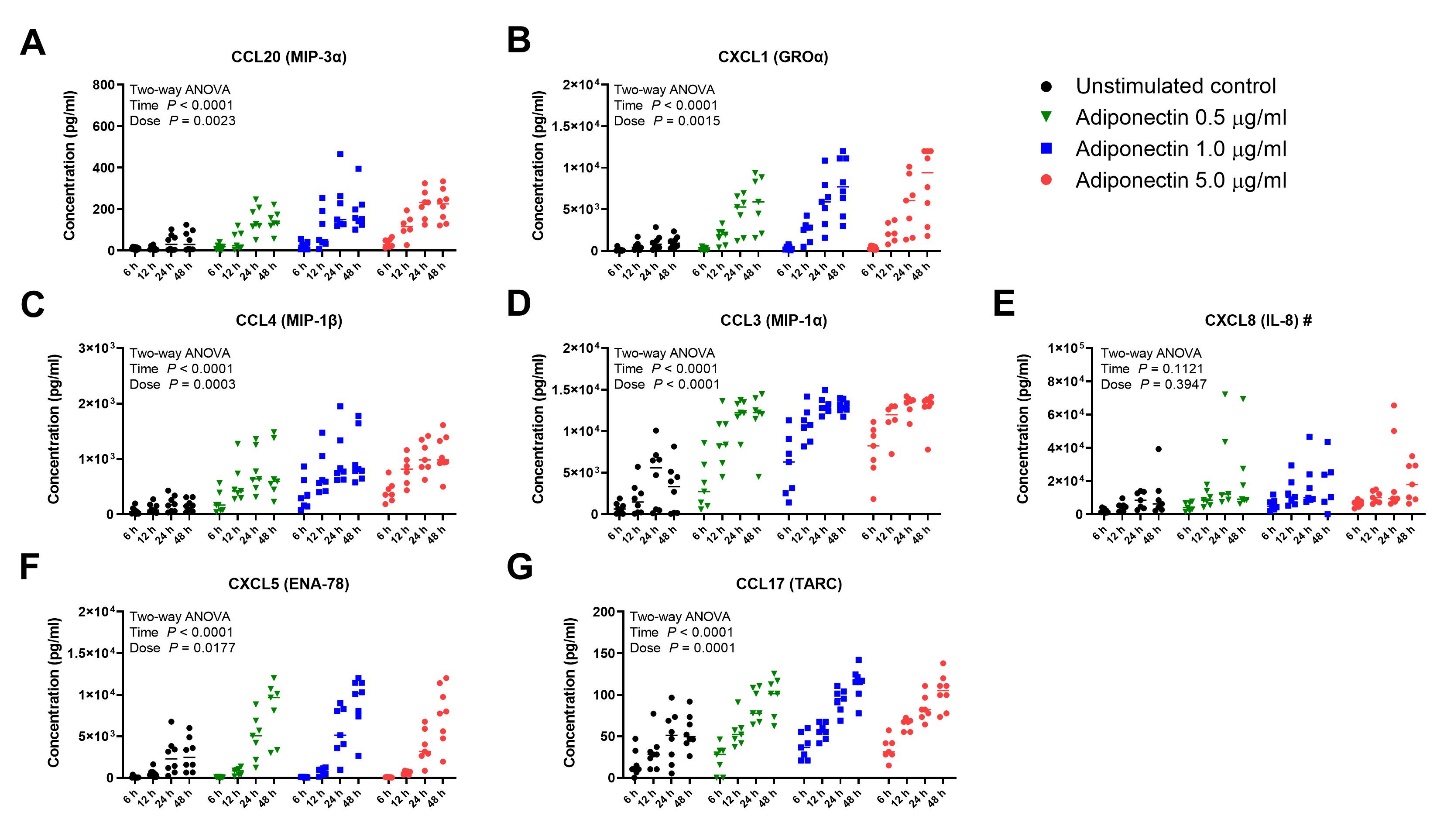


**Supplementary Figure 1.** **Time- and dose-dependent changes of chemokine levels in PBMCs from non-inflamed subjects.** Healthy PBMCs were stimulated by 0.5, 1.0 or 5.0 μg/ml adiponectin and the supernatants were collected at 6, 12, 24 or 48 hours after the stimulation. Levels of CCL20 (A), CXCL1 (B), CCL4 (C), CCL3 (D), CXCL5 (F), CCL17 (G) were measured using Legendplex assay. Levels of CXCL8 (E) were measured using ELISA (#). The results are shown as the individual values and medians from 7-8 healthy donors. *P* values were determined using two-way ANOVA assay.


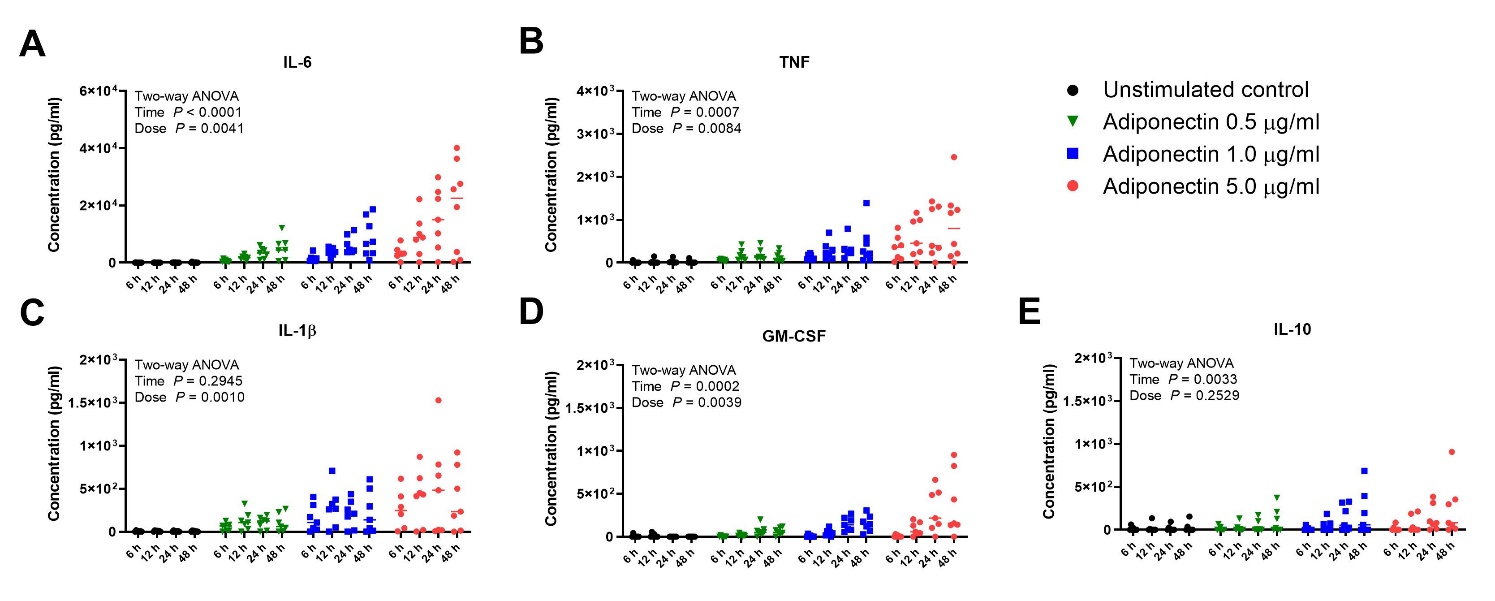


**Supplementary figure 2. Time- and dose-dependent changes of cytokine levels in PBMCs from non-inflamed subjects.** PBMCs were stimulated by 0.5, 1.0 or 5.0 μg/ml adiponectin, and the supernatants were collected at 6, 12, 24 or 48 hours after the stimulation. Levels of IL-6 (A), TNF (B), IL-1β (C), GM-CSF (D) and IL-10 (E) were measured using ELISA. The results are shown as the individual values and medians from 7-8 healthy donors. *P* values were determined using two-way ANOVA assay.

**
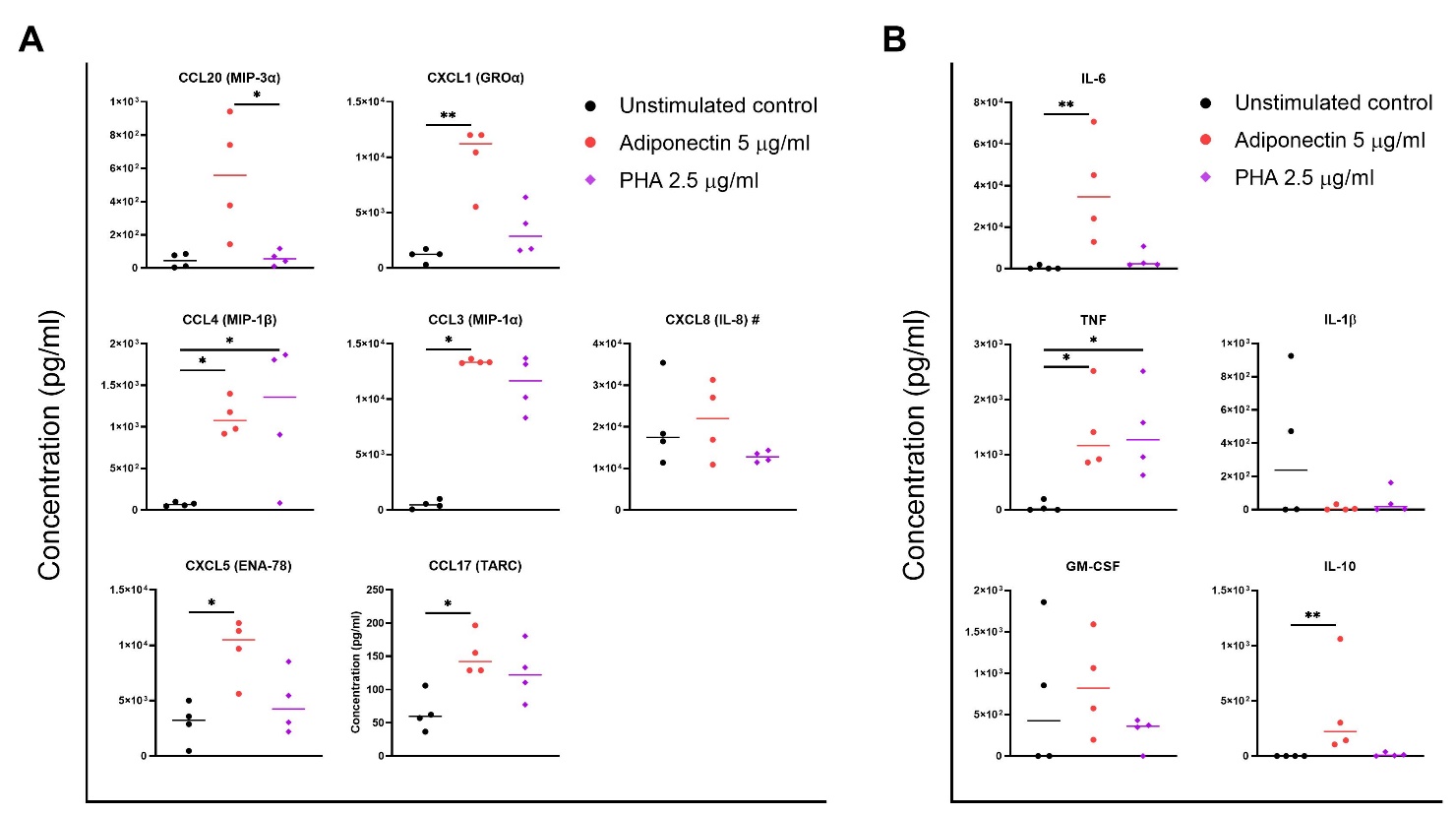
**

**Supplementary figure 3. Profile of chemokines and cytokines produced by adiponectin-stimulated PBMCs from subjects with RA.** Supernatants from unstimulated controls, adiponectin or PHA stimulated cells were collected from PBMCs at 48 hours after the stimulation. (A) Levels of CCL20, CXCL1, CCL4, CCL3, CXCL8, CXCL5 and CCL17 were measured using Legendplex assay or ELISA (#). (B) Levels of IL-6, TNF, IL-1β, GM-CSF and IL-10 were measured using ELISA. The results are shown as the individual values and medians from 4 subjects with RA. Significance was determined using Friedman’s test with post-hoc analysis unadjusted for multiple tests, **P* ≤ 0.05, ***P* ≤ 0.01.


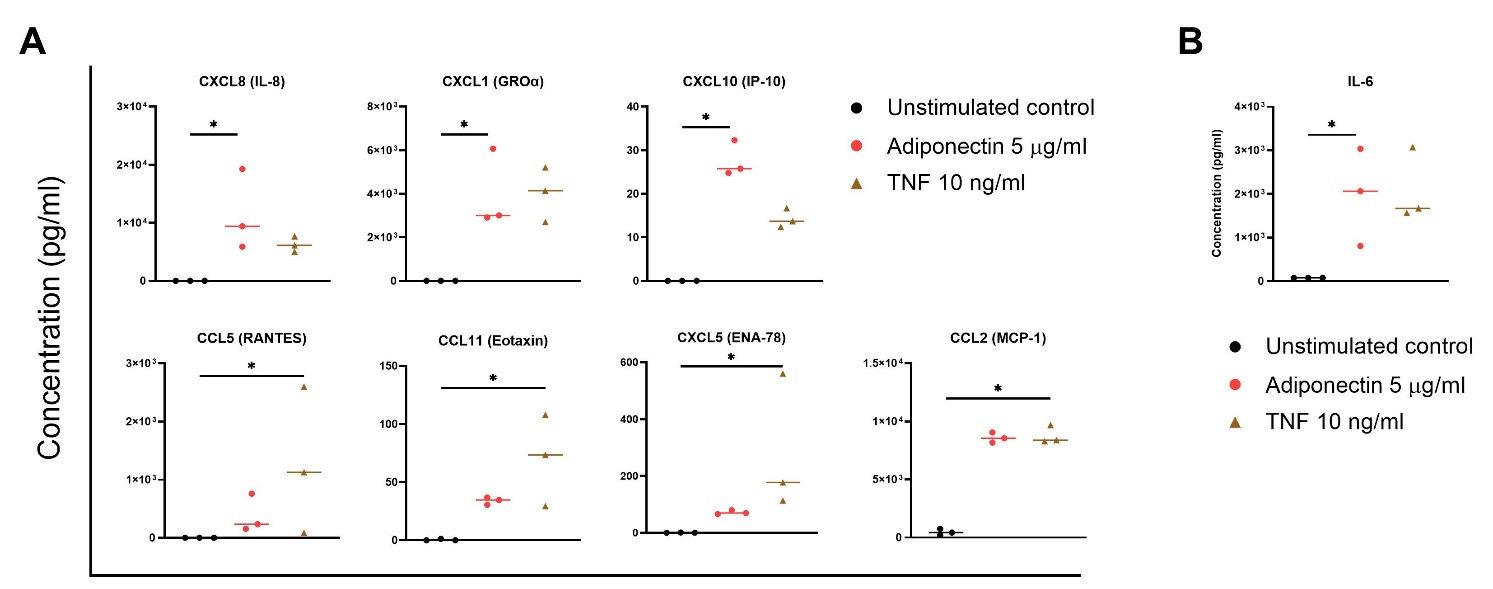


**Supplementary figure 4. Profile of chemokines and cytokines produced by adiponectin-stimulated FLS from subjects with RA.** Supernatants from unstimulated controls, adiponectin or TNF stimulated cells were collected from FLS at 48 hours after stimulation. (A) Levels of CXCL8, CXCL1, CXCL10, CCL5, CCL11, CXCL5 and CCL2 were measured using Legendplex assay. (B) Levels of IL-6 were measured using ELISA. The results are shown as the individual values and medians from 3 subjects with RA. Significance was determined using Friedman’s test with post-hoc analysis unadjusted for multiple tests, **P* ≤ 0.05.
